# Effects of process changes on emergency department crowding in a changing world: an interrupted time-series analysis

**DOI:** 10.1186/s12245-023-00479-z

**Published:** 2023-02-15

**Authors:** M. Christien Van Der Linden, Merel Van Loon-Van Gaalen, John R. Richards, Geesje Van Woerden, Naomi Van Der Linden

**Affiliations:** 1 Department of Emergency Medicine, Haaglanden Medical Center, P.O. Box 432, 2501 CK The Hague, the Netherlands; 2 Department of Emergency Medicine, University of California Davis Medical Center, PSSB 2100, 2315 Stockton Boulevard, Sacramento, CA 95817 USA; 3 Department of Health Technology and Services Research, Technical Medical Centre, University of Twente, P.O. Box 217, 7500 AE Enschede, the Netherlands

**Keywords:** Emergency department, Exit block, Patient flow, Length of stay, Surge capacity

## Abstract

**Background:**

During a 6-year period, several process changes were introduced at the emergency department (ED) to decrease crowding, such as the implementation of a general practitioner cooperative (GPC) and additional medical staff during peak hours. In this study, we assessed the effects of these process changes on three crowding measures: patients’ length of stay (LOS), the modified National ED OverCrowding Score (mNEDOCS), and exit block while taking into account changing external circumstances, such as the COVID-19 pandemic and centralization of acute care.

**Methods:**

We determined time points of the various interventions and external circumstances and built an interrupted time-series (ITS) model per outcome measure. We analyzed changes in level and trend before and after the selected time points using ARIMA modeling, to account for autocorrelation in the outcome measures.

**Results:**

Longer patients’ ED LOS was associated with more inpatient admissions and more urgent patients. The mNEDOCS decreased with the integration of the GPC and the expansion of the ED to 34 beds and increased with the closure of a neighboring ED and ICU. More exit blocks occurred when more patients with shortness of breath and more patients > 70 years of age presented to the ED. During the severe influenza wave of 2018–2019, patients’ ED LOS and the number of exit blocks increased.

**Conclusions:**

In the ongoing battle against ED crowding, it is pivotal to understand the effect of interventions, corrected for changing circumstances and patient and visit characteristics. In our ED, interventions which were associated with decreased crowding measures included the expansion of the ED with more beds and the integration of the GPC on the ED.

## Background

Emergency department (ED) crowding is a problem in many countries [1]. Negative consequences of crowding include delayed patient care and poorer outcomes for patients [2–5]. The major contributor for crowding is the lack of hospital capacity, which leads to admitted patients boarding in the ED [4]. As such, crowding is not an issue isolated to the ED but a hospital-wide, systemic issue [5]. Boarding reduces the quality of care [2, 6] and increases the duration of hospital stays [2, 7, 8]. Although many factors contributing to crowding are external to the ED, there are multiple options for process improvement during the input, throughput, and output phases of the ED [9]. Researchers have evaluated multiple interventions intended to reduce crowding, varying from the implementation of acute admission units to Lean projects, in different ED settings [2, 4]. Often, these studies concern the effect of small-scale solutions to either input, throughput, or output issues. The numerous interventions proposed to reduce crowding show the complexity of the problem. Crowding research often fails to take changing circumstances, ED populations, and external influences into account or fails to separate the effects of each intervention on crowding when multiple interventions are introduced in a limited period. Controlled studies looking at crowding outcomes during infectious disease outbreaks, such as influenza and the COVID-19 pandemic, are scarce.

In this study, we assess the effects of process changes that we introduced during a 6-year period. The process changes, such as the introduction of a general practitioner cooperative (GPC) at the ED and additional medical staff during peak hours, were expected to improve patient flow and decrease crowding. Meanwhile, centralization of emergency care took place, decreasing the number of EDs from three to one. We aim to provide insight into the effects of several process changes on crowding, patients’ ED length of stay (LOS), and the number of exit blocks in the remaining ED, while accounting for changes in external circumstances and a changing population using a time-series design.

## Methods

### Study design

We assessed the effects of several process changes and external circumstances over a 6-year period (August 2014 to August 2020). Using a time-series design, we described the effects on three crowding measures: ED patients’ LOS, a modified version of the National ED Overcrowding Scale (mNEDOCS), and the number of patients experiencing exit block. Exit block was defined as LOS longer than 4 h for a patient requiring hospital admission, based on the 4-h rule [10]. We extracted the following patient and visit characteristics from the hospital’s database for each registered patient: age, sex, presenting problem, triage level, and day and time of the visit. Presenting problems were based on the triage flow charts chosen by the triage nurse. Presenting problems occurring less than 500 times per year were categorized as “Other” and included allergy, diabetes, exposure to chemicals, fits, foreign body, irritable child, pregnancy, rashes, sexually acquired infections, and worried parent. Triage levels were assigned according to the five-level Manchester Triage System [11].

### Study setting

Haaglanden Medical Center (HMC) is located in The Hague, the Netherlands, home to more than 500,000 people. HMC delivers hospital care at three hospitals: Antoniushove, Bronovo, and Westeinde. Acute care was centralized from three EDs to two in 2017 and from two EDs to one in 2019. The remaining 34-bed ED (Westeinde) serves as a regional level 1 trauma and acute neurovascular center and has a 29% admission rate.

The usual staffing at this ED includes an emergency physician (EP) attending and EP residents 24 h per day, 7 days per week. Residents of cardiology, neurology, surgery, and internal medicine are available in-house 24/7. The nursing staff consists of certified emergency nurses (75%), nurse practitioners (NPs) (5%), and registered nurses in training for the emergency nurse (20%).

### Process changes during the 6-year study period

#### Pilot presence of medical specialists at the ED

During 8 weeks in 2016 (October 3 until November 27), five medical specialists (cardiology, internal medicine, neurology, radiology, and surgery) were present at the Westeinde ED during weekdays between 17:00 and 23:00 pm and during the weekends between 14:00 and 18:00 pm. The medical specialists performed direct supervision or a combination of direct and indirect supervision on the EP-led ED. Analyses showed improvement of the throughput of admitted patients and patient satisfaction [12, 13]. Based on these pilot results, changes in staffing were implemented in November 2017 (see below).

#### Centralization of emergency care

In April 2017, HMC closed one of three separate EDs (Antoniushove). In preparation for the closure of the second ED (Bronovo), HMC first closed the four-bed intensive care unit (ICU) at Bronovo in October 2018, relocating the beds, equipment, and staff to the ICU at Westeinde. The Bronovo ED closed its doors in July 2019, centralizing emergency care into one site (Westeinde). Prior to this centralization, the three EDs were fully functional with average annual ED volumes of 15,512 (Antoniushove, 2016), 28,702 (Bronovo, 2018), and 57,718 (Westeinde, 2018). In the remaining ED (Westeinde), the implementation of several system changes occurred simultaneously with the closure of Bronovo to mitigate the potential negative effects of the centralization. System changes included streaming eligible patients directly from triage to dedicated minor injury units, establishment of a cardiac care decision unit, and service-level agreements with inpatient hospital departments to improve the outflow of admitted patients. In addition, we shifted next-day check-up appointments for discharged ED patients to the outpatient clinics. Moreover, the ED staff of Bronovo started working at the ED of Westeinde. The intention of all changes was to prevent an increase in crowding due to higher patient numbers.

#### Psychiatry project

During a 9-month period (May 2017 until February 2018), a psychiatric intervention team was added to the usual psychiatric care at the Westeinde ED. Previously, there was only one psychiatric intervention team available for the entire city that came to the ED to assess patients with mental health problems. For this project, a psychiatric intervention team was physically present from 15:30 pm to midnight, aiming to reduce waiting times and LOS of patients with mental health problems. While the number of patients with mental health problems increased during the study period, patients’ LOS decreased significantly [14]. Due to a lack of funding, the project ended after 9 months.

#### Pilot Lean-driven interventions and dedicated ED radiologist

In September 2017, the radiology department organized a 5-day Lean project to identify bottlenecks throughout the imaging process at the ED. Subsequently, the team implemented several Lean strategies, such as a diagnostic fast track for computed tomography (CT), and agreements on communication by telephone to prevent unnecessary calls. In addition, we added a dedicated radiologist to the ED during peak hours. Radiology turnaround times and report times for ED patients decreased significantly [15]. Based on the positive pilot results, the intervention was continued to become standard practice.

#### Changes in staffing

In November 2017, we added one emergency NP to the team each day and evening shift to improve throughput for patients with low-acuity complaints who were ineligible for redirection to the GPC. We also expanded the ED medical staff, based on the 2016 pilot results [12]. During peak hours at the ED (from noon until 20:00 pm), a cardiologist, an internal medicine specialist, a neurologist, a radiologist, and a surgeon were physically present at the ED, performing direct on-site supervision. They made additional notes in the medical records themselves if necessary. They worked side-by-side with the EP attending and residents and did not have any other tasks in the outpatient clinic or clinic during their ED service. The coordinating EP was able to initiate a team-based approach when indicated, with patients assessed and managed simultaneously by attending specialists at arrival. Other medical specialists were available in the hospital (office hours) and on-call (out-of-office hours) when consulted. Duration of high mNEDOCS scores decreased significantly, as did patients’ ED LOS. However, we did not control for other influences in the analyses [16]. During the COVID-19 peak, staffing was adapted again. A pulmonologist was available at the ED from 17:00 to 20:00 pm. Moreover, the ED deployed two extra nurses per day shift and evening shift.

#### General practitioner cooperative

From 2013 until January 2020, a GPC was located on the hospital site with a separate entrance, 100 m away from the Westeinde ED. The ED triage nurse could stream part of the self-referrals to the GPC based on a triage assessment. This led to an efficient redirection of self-referrals but failed to improve the throughput of the remaining patients at the ED [17]. In February 2020, further integration of the GPC within the ED took place into a primary care service with open radiological access. Nowadays, staff from ED and GPC work side by side in the reception area. For self-referred patients, GPC receptionists use screening questions to decide on the eligibility of assessment at the GPC. The GPC staff registers the self-referred patients with primary healthcare problems, and the ED staff registers the remaining patients in the hospital system. The GPC is open from 8:00 am to midnight, 7 days a week. During the night, patients who fail to consult their own GP or out-of-hours GP service are registered as self-referrals.

### External circumstances during the study period

#### Influenza season

Each year, the influenza season during the cold winter months exposes EDs to surges in demand, mostly by patients aged 65 years and older who require hospital admission [18, 19]. This may cause an exit block. The duration of the influenza season in the Netherlands varies per year. We included the number of weeks of the influenza seasons of 2015 (21 weeks), 2016 (11 weeks), 2017 (15 weeks), 2018 (18 weeks), and 2019 (14 weeks), based on the reports of the Dutch Centre for Infectious Disease Control [20]. In 2020, the influenza epidemic was relatively mild and short (5 weeks) and partly overlapped with the COVID-19 pandemic.

#### COVID-19 pandemic

In the Netherlands, the first COVID-19 case was confirmed on February 27, 2020. We included the period of March 16 to May 26, 2020, as the COVID-19 peak in the study setting. During this period, the Dutch government announced social distancing rules and banned large public events and gatherings. Schools and day cares were closed, except for children whose parents worked in the “vital” sectors, like health care. People were urged to work from home.

### Data collection and analysis

ED crowding was measured with a modified version of the NEDOCS [21, 22], a multidimensional scale. Variables needed to calculate the NEDOCS include the total number of ED beds, total hospital beds, total patients in the ED, total admits in the ED, longest admit time, and waiting room time of the most recent patient placed in a bed in the ED. Furthermore, the number of respirators in use is needed to calculate the NEDOCS, but since these are not registered in the hospital information system, we used the number of patients who are being resuscitated or assigned the highest acuity level, leading to the mNEDOCS. These variables are derived automatically from the hospital information system every 15 min, 24/7. The mNEDOCS has been previously shown to correlate well with perceived crowding in this ED [22]. Crowding is defined as a mNEDOCS of > 100.

We calculated ED LOS (registration to departure) and the number of patients experiencing exit block (LOS of more than 4 h for patients who need hospital admission) for all patient visits during the study period. We summarized mNEDOCS scores, LOS, and the number of exit blocks per week and determined time points (weeks) at which the various interventions and external circumstances took effect.

We built an interrupted time-series (ITS) model per outcome measure using ARIMA modeling to account for autocorrelation in the outcome measures. In the base case analyses, we selected the best fitting ARIMA model per outcome measure, using the “Expert Modeler”-module in SPSS. Stationary *R*-squared was used as the primary goodness-of-fit measure. The time unit used in the models is weeks.

The Statistical Package for the Social Sciences [IBM Corp., IBM SPSS Statistics for Windows, version 28.0.1.0 Armonk, New York, USA] was used for the analyses. The regional medical research ethics committee exempted the study (METC-LDD, G20-082).

## Results

**Fig. 1:**
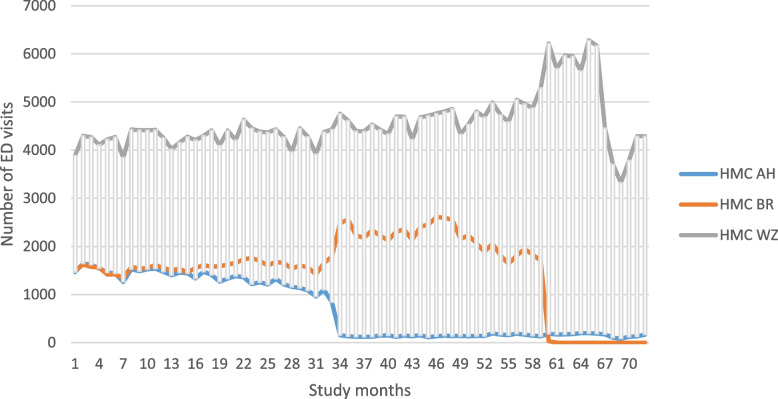
Number of emergency department visits during the 6-year study period. Abbreviations: ED, emergency department; HMC AH, Haaglanden Medical Center Antoniushove; HMC BR, Haaglanden Medical Center Bronovo; HMC WZ, Haaglanden Medical Center Westeinde

During the study period, 487,375 ED visits were registered: 50,201 in Antoniushove (ED closure in month 33), 108,802 in Bronovo (ED closure in month 60), and 318,372 in Westeinde (Fig. 1).

**Fig. 2:**
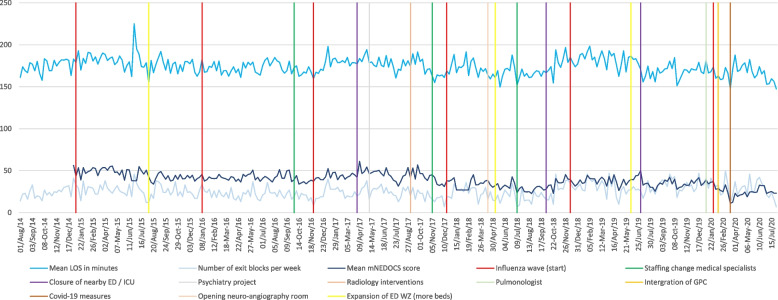
Outcome measures over time, including main interventions and external circumstances. Abbreviations: ED, emergency department; GPC, general practitioner cooperative; ICU, intensive care unit; LOS, length of stay; mNEDOCS, Modified National ED OverCrowding Score; WZ, Haaglanden Medical Center Westeinde

Figure 2 shows the sum of the crowding measures over time, including the main interventions and the external circumstances.

### Effects of the process changes

Patients’ ED LOS decreased with the expansion of the ED from 22 to 26 beds (Table 1). After the closure of the ED of a neighboring ED in 2017, we observed an increase of the mNEDOCS. The mNEDOCS decreased significantly when another eight beds were added to the ED, as well as with the further integration of the GPC at the remaining ED. After the closure of the ICU at another neighboring hospital in 2019, we observed an increase in the number of exit blocks at the remaining ED.

**Table 1:** ARIMA model specification for outcome measures “mean length of stay,” “mean mNEDOCS,” and “sum of exit block”

		Estimate	SE	Significance
Length of stay	MA Lag 1	0.793	0.37	<0.001
Interventions				
Expansion of the ED to 26 beds	Numerator Lag 0	−16.078	6.297	0.011
	Denominator Lag 1	−0.685	0.261	0.009
External circumstances				
Influenza 2018–2019	Numerator Lag 0	8.222	3.373	0.015
Patient and visit characteristics				
Admission	Numerator Lag 0	0.187	0.26	<0.001
	Denominator Lag 1	0.375	0.108	<0.001
Back pain	Numerator Lag 0	−0.363	0.125	0.004
Ear, nose, throat, and eye problems	Numerator Lag 0	−0.347	0.094	<0.001
Extremity problems	Numerator Lag 0	0.043	0.021	0.045
Other presenting problems	Numerator Lag 0	−0.124	0.048	0.010
Urgent (yellow/orange) triage category	Numerator Lag 0	0.058	0.029	0.044
Daytime	Numerator Lag 0	−0.061	0.018	<0.001
mNEDOCS	MA Lag 1	0.611	0.048	<0.001
Interventions				
Integration GPC	Numerator Lag 0	−12.021	3.929	0.002
Expansion of the ED to 34 beds	Numerator Lag 1	−11.998	3.971	0.003
External circumstances				
Closure ED HMC AH	Numerator Lag 0	17.318	4.946	<0.001
	Numerator Lag 1	12.610	4.946	0.011
Exit block	MA Lag 1	0.888	0.029	<0.001
External circumstances				
Closure of ICU at HMC BR	Numerator Lag 0	20.460	6.235	0.001
	Numerator Lag 1	17.763	6.295	0.005
Influenza 2018–2019	Numerator Lag 0	7.973	2.293	0.001
Patient and visit characteristics				
Admission	Numerator Lag 0	0.144	0.019	<0.001
Shortness of breath	Numerator Lag 0	0.089	0.033	0.007
Unwell patient	Numerator Lag 0	−0.083	0.029	0.005
Age 70 years and older	Numerator Lag 0	0.090	0.019	<0.001
Daytime	Numerator Lag 0	−0.029	−2.614	0.009

*Abbreviations*: *ARIMA*, autoregressive integrated moving average; *ED*, emergency department; *GPC*, general practitioner cooperative; *HMC AH*, Haaglanden Medical Center Antoniushove; *HMC BR*, Haaglanden Medical Center Bronovo; *ICU*, intensive care unit; *mNEDOCS*, modified National Emergency Department OverCrowding Score; *SE*, standard error

### Effects of external circumstances

The severe influenza wave of 2018–2019 was associated with increased patients’ ED LOS and with exit block.

### Patient and visit attributes

Patients’ ED LOS increased with more inpatient admissions, more urgent patients, and when more patients arrived with extremity problems. We observed a shorter LOS during daytime and when more patients arrived with back pain, ear, nose, throat, or eye problems, or with “other” presenting problems (rest category). There were more exit blocks with more patient admissions, more patients presenting with shortness of breath, and more patients 70 years or older. There were less exit blocks during daytime and when more “unwell” patients registered at the ED.

## Discussion

Our study reflects the need for continuously improving ED flow by anticipating changing circumstances and populations. Our results show that it is challenging to improve the multifactorial problem of crowding during challenging conditions such as pandemics, centralization, and a changing population. The effects of most of the interventions we implemented to decrease crowding and improve patient flow were not picked up as statistically significant in the models, while we have reported positive findings of these interventions in previous studies. For example, in our before and after intervention study concerning the physical presence of a psychiatric intervention team, we showed a 46-min decrease in ED LOS for patients with psychiatric problems [14]. In our before and after study concerning additional staffing of medical specialists during peak hours and extended opening hours of the admission office, we demonstrated significantly less mNEDOCS measurements above 100 (indicating crowding) during the intervention period [16]. In the present study, we found no effect of these costly interventions on our crowding measures in the total ED population when corrected for confounding factors. Various reasons for this may exist. For example, some interventions may particularly impact selective subgroups of the total ED population, while our analyses did not stratify by subgroup. Also, Dutch LOS is already short by international standards [23], making it difficult to realize clinically relevant reductions.

Experts agree that process improvements limited solely to the ED will not solve crowding [2, 24] since the fundamental problem is the lack of hospital capacity [2, 25]. This may explain the lack of effect of our interventions on the three crowding measures: most of our interventions were not typical “outflow” interventions. The interventions that affected our crowding measures were no outflow interventions, and their effect should be interpreted with caution. While the present study shows that implementing a GPC decreased the mNEDOCS, this is hardly surprising since all self-referrals were diverted to the GPC, causing a dramatic decrease in the number of ED visits. Since the mNEDOCS includes the number of ED visits, the mNEDOCS obviously decreased. The implementation of the GPC had no effect on the remaining patients’ ED LOS, supporting the widespread belief that diverting low-complexity patients is unlikely to reduce the ED LOS of the remaining patients [26, 27]. In a previous study, we showed that the introduction of a GPC led to an efficient redirection of self-referrals but failed to improve the throughput of the remaining ED patients [17]. The further integration of the GPC seems to have the same effects. Other interventions that affected our crowding measures were expanding the number of ED beds, which decreased LOS and mNEDOCS. Again, the latter is not surprising, since the number of ED beds is part of the mNEDOCS score. ED expansion alone is not an adequate solution to ED crowding [28], but probably our staffing resources matched service demands.

The present study shows that external circumstances as well as patient and visit characteristics affect crowding most. The closure of the first neighboring ED in April 2017 caused an increase in the mNEDOCS in Westeinde, coinciding with the study of El Sayed et al. [29].

Schull et al. [19] showed that the association between influenza season and ED crowding is independent of an increase in total ED visits, suggesting that worsened crowding primarily is the result of an increased utilization by elderly patients with major respiratory illnesses. This supports the widespread belief that exit block from lack of inpatient hospital capacity is the single most important cause for ED crowding. During our 6-year study period, six influenza seasons occurred, ranging from a 21-week period (2015) to a 5-week period (2020). In 2016, our hospital installed a dedicated team to increase the availability of hospital beds during influenza season to cope with the predictable need. A dedicated influenza team orders to delay planned care to increase hospital capacity for acute care, based on daily incident case counts. This may explain the lack of effect of the influenza seasons on crowding in our hospital. The 2018–2019 influenza season was unexpectedly long, resulting in increased LOS and number of exit blocks.

In the weeks before the first peak of the COVID-19 outbreak in the Netherlands, we anticipated substantial crowding due to high patient numbers and increased hospitalization needs. Forewarned by the Wuhan, China, and Lombardy, Italy, experiences [30–32], we initiated preparations such as staff training, triage protocols, personal protective equipment orders, and reconfiguration of the hospital. We installed special admission units for COVID-19-suspected patients, additional ED and ICU staffing, the GPC as an alternative site, and an extra CT scan at the ED. Meanwhile, a record low number of patients with non-COVID-19-related complaints was noticed, probably due to the regulations (stay-at-home policy, travel restrictions, working remotely), the hospital delaying planned care, and patients avoiding the hospital out of fear of contracting the virus. Another possible reason for the decrease in ED presentations was an absolute decrease in infectious diseases due to the prevention policies for COVID-19. During the last week that schools were open in our country, the GPs registered 70–80% less children under 4 years of age with infectious diseases such as ear infections, eye infections, and impetigo [33]. This is probably due to hand washing, social distancing, and stay-at-home policies. For medical conditions not transmitted through human contact, such as hay fever, no decline in GP registrations was noted [33]. The prevention policies may have led to fewer infectious diseases in adults too and explain the relatively smaller influenza outbreak compared to other years. During the COVID-19 pandemic, we did not observe an increase in our crowding measures. This occurred in more EDs in the world: a consistent respite from crowding was observed in many EDs across North America [34]. As many of the COVID-19-suspected patients needed admission, the hospital opened special admission units and extra ICU beds during the preparation period.

### Strengths and limitations

Our study presents a long longitudinal series of data (6 years) including all ED visits, and patient and visit characteristics, allowing us to evaluate changes in the ED population accurately. Moreover, we measured crowding using three measures, among which the mNEDOCS scores almost every 15 min of these 6 years. Although none of the crowding metrics is without limitations [5], the mNEDOCS was previously validated in our study setting [22], hence providing an accurate estimate of ED crowding over the 6-year study period. For the COVID-19 peak, we included a 9-week period, until May 19, 2020. After that period, the Dutch government loosened restrictions, partly opening schools, restaurants, and indoor pools, provided that no new outbreaks would occur. The data collection ended well before the second COVID-19 wave hit the Netherlands, in September 2020.

One source of uncertainty stems from the use of outcome variables which are partly overlapping and for which no gold standard exists. For example, while we defined exit block as ED LOS of 4 h or more for patients needing hospital admission, for some jurisdictions, an ED LOS of 8 h is used [35].

Precise modeling of the effects of the process changes and external circumstances is challenging because no modeling approach fully captures the complexities of the ED system [36]. Our ITS statistical technique may accurately evaluate changes in the outcome measures but does not allow cause-effect conclusions.

While ARIMA models are commonly used in ITS analyses, other statistical models can be used for this purpose (e.g., generalized least squares, restricted maximum likelihood), each with their own pros and cons. One advantage of ARIMA is that it “explicitly models the influence of previous time points by including regression coefficients from lagged values of the dependent variable and errors” [37, 38]. No comparison was made between (findings from) alternative models, so the impact of the modeling technique on our outcomes has not been objectified.

Sometimes it takes a while before an intervention is running as intended, so delayed effects are reasonable. Some interventions overlapped, e.g., the Lean radiology project started before ending the psychiatry pilot. In case of delayed effects, the impact of individual interventions or circumstances may have been over- or underestimated. In addition, it was impossible to capture all process changes in this 6-year study period, because many of the smaller changes are not registered, published, or assessed separately from other changes. A number of small process changes occurred which may have had effects on our outcome variables, such as assigning an additional nurse to triage during peak hours, redesigning physician shift schedules to better align with patient volumes, and using nurse order protocols in triage. The latter enables the triage nurse to order laboratory and radiology tests earlier in the process. In the present study, we mainly included process changes that we previously assessed using quasi-experimental (before-and-after) studies [12–16] and external circumstances as registered by the National Institute for Health Services Research.

Findings between the prior before-and-after studies and the current study differ. This may be due to using a more robust method in the current study, while including external circumstances and measured effects over a larger population and a longer period of time. For example, the before-and-after study on the psychiatry project showed a 46-min decrease in LOS for patients with a mental health problem. However, these patients only reflect 3% of our total ED visits. In the present study, effects in such a minor subpopulation might not have been picked up, despite potential clinical relevance in the subgroup.

Multiple of our interventions did not result in an overall improvement of our crowding measures. Future studies are needed to assess other effects, such as an improvement of quality of care and/or effects on workload. For some of our interventions, there was a large investment in extra staffing. The ratio between investment and returns has not been investigated in this study.

The generalizability of our study may be limited to similar EDs with relatively short LOSs.

## Conclusion

A number of process changes and external circumstances were associated with crowding measures, both negatively and positively. Our findings reflect the importance of the ongoing battle against ED crowding and the need to continuously identify bottlenecks and improve ED flow, anticipating changing circumstances and populations. Ongoing and timely feedback on new interventions is vital to increasing the success and sustainability of projects, and long-term effects corrected for population changes provide some lessons in terms of which interventions to prioritize and how to best improve ED processes and patient care.

## Data Availability

The datasets used and analyzed during the current study are available from the corresponding author on reasonable request.

## Abbreviations

CI: Confidence interval
CT: Computer tomography
ED: Emergency department
EP: Emergency physician
Et al.: et alii, meaning “and others”
*n*: Number
NP: Nurse practitioner
EP: Emergency physician
GP: General practitioner
GPC: General practitioner cooperative
HMC: Haaglanden Medical Center
ICU: Intensive care unit
IQR: Interquartile range
ITS: Interrupted time-series
LOS: Length of stay
mNEDOCS: Modified National Emergency Department OverCrowding Score
NEDOCS: National Emergency Department OverCrowding Score
SPSS: Statistical Package for the Social Sciences
